# An evaluation of systematized phonics on reading proficiency in Swedish second grade poor readers: Effects on pseudoword and sight word reading skills

**DOI:** 10.1002/dys.1669

**Published:** 2020-09-28

**Authors:** Maria Levlin, Cecilia Nakeva von Mentzer

**Affiliations:** ^1^ Department of Language Studies Umeå University Umeå Sweden; ^2^ School of Health Sciences Örebro University Örebro Sweden

**Keywords:** policy change, reading difficulties, response to intervention, systematized phonics

## Abstract

The aim of the present study was to evaluate the effect of systematized phonics on word reading in Swedish second grade poor readers. Forty‐nine children who performed at or below the 25th percentile on pseudoword reading and/or sight word reading at the beginning of second grade participated in the study. The study had a cross‐over design exploring within‐and between‐group effects of two different conditions: systematized phonics and classroom instruction. Overall, systematized phonics proved more effective than classroom instruction. At pre‐intervention, no child performed above the 30th percentile in pseudoword reading or sight word reading. At post‐intervention, corresponding numbers were 69% for pseudoword reading and 35% for sight word reading. Implications for a policy change in Sweden towards mandatory systematized phonics in primary school are discussed.

## 1 INTRODUCTION

In Sweden, formal reading tuition begins when the child starts school at 7 years of age. According to the Swedish National Agency of Education (2019), for the first three school years, reading tuition should mainly focus on the alphabet, phoneme–grapheme correspondence and reading strategies for comprehension and decoding. However, very few explicit guidelines are provided on how this should be accomplished, and there are few instructions regarding which reading methods are effective and how they should be taught. In typical reading development, the child reaches the orthographic reading stage (Frith's model of reading acquisition, 1985; logographic, alphabetic and orthographic) at the end of the second school year (Herrlin & Lundberg, 2014). Orthographic reading is accomplished through the amalgamation of orthographic and phonological representations (West, 2000) resulting in a rich orthographic lexicon, an essential prerequisite for fluent reading (Rakhlin, Mourgues, Cardoso‐Martins, Kornev, & Grigorenko, 2019). As a consequence of orthographic reading, tuition shifts from learning to read to *reading to learn*, where reading becomes an important tool for knowledge acquisition and educational outcome (Conti‐Ramsden, Durkin, Simkin, & Knox, 2009; Dockrell, Lindsay, & Palikara, 2011). However, for struggling readers, orthographic reading is challenging (Van der Kleij, Segers, Groen, & Verhoeven, 2019), which leads to long‐term negative personal and societal effects (Hakkarainen, Holopainen, & Savolainen, 2013; Kiuru et al., 2011; Smart et al., 2017). Consequently, preventing these negative consequences is critical.

### 1.1 Early reading instruction

Thirty years ago, the Bornholm study (Lundberg, Frost, & Petersen, 1988; Lundberg, Rydkvist, & Strid, 2018) in which preschool children received systematized phonological awareness‐training, showed positive effects on reading and spelling skills in early school years. The training was particularly effective at improving the scores of poor performers. Nowadays, this highly systematic intervention is an essential part in the Swedish preschool curriculum (Swedish National Agency of Education, 2017). However, analogous systematic reading intervention for young struggling readers is still not implemented nationally in Sweden, with negative consequences for children at risk of developing reading difficulties. Recent investigations (Teachers' National Association, 2013) report a lack of specific education in reading methods for teachers at primary school level. Furthermore, the national curriculum (Swedish National Agency of Education, 2019) provides no explicit guidance on early decoding instruction. Only 2 of the 24 curriculum bullet points address this issue, with the rest instead mainly focusing on strategies for reading comprehension and text composition.

In order to diminish the gap between research findings on effective reading methods and educational policy and practice, Castles, Rastle, and Nation (2018) presented a comprehensive tutorial review of the science of learning to read. The tutorial starts by stressing the importance of young learners cracking the alphabetical principle in alphabetic writing systems (e.g., English and Swedish). Second, it describes the positive effects of implementing systematized phonics instruction on broader literacy performance, both short‐term (2 years after phonics instruction) and long‐term (6 years after phonics instruction). Particular advantage was observed for children who had a high probability of starting school as struggling readers. A large number of studies show similar positive outcomes of systematized phonics (Blachman et al., 2014; Gustafson, Fälth, Svensson, Tjus, & Heimann, 2011; McArthur et al., 2015; Nakeva von Mentzer et al., 2013; Vellutino, Scanlon, Zhang, & Schatschneider, 2008; Wanzek et al., 2018; Wolff, 2011). A meta‐review by Wanzek and Vaughn (2007) presented some benefits of a one‐to‐one setting over small groups. While phonics has been embedded in educational recommendations in both the United States (National Institute of Child Health and Human Development, 2000) and the United Kingdom (Wyse & Styles, 2007), it has not been implemented in Sweden to the same degree (SBU, 2014).

There have been efforts to realize intense reading intervention at the municipality level in Sweden. For example, Wolff (2011, 2016) combined phoneme–grapheme mapping, reading comprehension and reading speed in an intervention to Grade 3‐poor readers. The children received 40 hr of training in a one‐to‐one setting, conducted daily in schools over 12 weeks. Wolff proved that reading comprehension skills, spelling, phonemic awareness (PA) and reading speed can be enhanced, with positive effects remaining one year later. In the 5‐year follow‐up (Wolff, 2016), effects were observed only on word decoding, although a broad spectrum of reading tasks comprised the intervention, making it difficult to determine what feature contributed to this effect. In another Swedish study (Gustafson et al., 2011), computer‐based reading intervention (bottom‐up, top‐down or a combination of both) was performed at the municipality level for struggling second grade readers. After 7–8 hr of training, improved reading skills were reported. However, no information regarding how many children reached age‐adequate reading skills was presented in either study. This information is essential in understanding whether these children may reach the *reading to learn* level after intervention.

In light of this, the present study was launched to explore the possible effects of an intensive one‐to‐one 6‐week systematized phonics instruction in children identified as struggling readers in the beginning of Grade 2, and to what extent age‐typical word reading skills were reached.

### 1.2 The present study

The present study was part of a project in the municipality aimed at implementing and evaluating new routines for early identification and support for struggling readers. The training included phoneme–grapheme correspondence, PA and word recognition elements. In the present study, word recognition included blending phonemes into words by decoding single words. Two different materials were used in word recognition training, *Bravkod* (‘good decoding skills’; Ingvar, 2008; Jönsson, 2010) and *Trugs* (‘teach reading using games’; Häggström & Frylmark, 2010; Jeffrey, 2018). Both materials are commonly used within special needs education for struggling readers in Sweden, but neither of them has been systematically evaluated previously in a controlled trial, individually or in combination. Consequently, the present study fills an important gap in the Swedish reading intervention context. The aim of the present study was to evaluate the effect of systematized phonics on word reading proficiency in a semi‐transparent orthography (Swedish) for a group of struggling second grade readers in a Swedish educational setting. Our research questions were:

1. What are the effects of systematized phonics on word reading in a group of children identified as poor readers in the beginning of grade 2?
2. To what extent does systematized phonics support reaching age‐adequate word reading skills?

## 2 METHOD

### 2.1 Participants

A total of 267 children from nine different schools participated in a group assessment of word reading (Jacobson, 2014) and reading comprehension (Järpsten, 2004) at the end of Grade 1, after 1 year of formal reading instruction. Children performing 1.0 standard deviations below the mean at the end of Grade 1 (see Table 1) participated in an individual assessment at the start of the first semester in Grade 2 (*n* = 85). Children performing at or below the 25th percentile in pseudoword reading and/or sight word reading in the individual assessment in Grade 2 were invited to participate in the current study (*n* = 57). Seven children were not able to participate in the assessment in Grade 1, but were included in the individual assessment in Grade 2 due to their teachers' concern about their reading development. All parents of the 57 children were informed about the study and 49 families gave their written consent to participate in the study.

**TABLE 1:** Screening scores in reading for enrolled children in Grade 1

Screening in grade 1		Raw scores		Z‐scores	
	N	M	*SD*	M	*SD*
Word reading	42	3.00	2.25	−1.15	0.35
Reading comprehension	44	6.82	3.60	−1.65	0.83

*Note*: Initial reading performance scores are missing for seven children in word reading and for five of them also in reading comprehension.

The mean age was 8.1 years (min = 7.6, max = 9.3 years) for all participants. Eight children (two girls and six boys) had Swedish as a second language (SSL). Five of these children had lived in Sweden less than 2 years. It was not possible to match the groups for gender and SSL. Thus, there was a higher proportion of boys in Group 1 and a higher proportion of children with SSL in Group 2 (see Table 2). The assignment to Group 1 (*n* = 22) or Group 2 (*n* = 27), respectively, was stratified, that is, the project leader and the first author identified children with severe, moderate and mild word reading difficulties (in relation to the percentile‐score in pseudoword and sight word reading at T1) and students from each category were assigned to Group 1 or Group 2. However, due to organizational circumstances at some of the schools, the categorization was not completely randomized.

**TABLE 2:** Demographic data by group

	Total				Group 1				Group 2			
	*n*	M	*SD*	%	*n*	M	*SD*	%	*n*	M	*SD*	%
Age (months)	49	97	4.1		22	97	3.9		27	97	4.4	
Sex (% female)				41				32				48
SSL				16				4				26

Abbreviation: SSL, Swedish as a second language.

### 2.2 Test procedure

The group assessment (word reading and reading comprehension) at the end of Grade 1 was conducted by the teacher or specialist education teacher in the classroom. This was part of the regular procedure in the schools in this municipality. The individual assessments (T1, T2 and T3) and all scorings in Grade 2 were conducted by a specialist education teacher responsible for the reading assessments at the School Health Services (also project leader in the municipality). The administration of the tests followed the standard procedures in the manuals.

Three individual assessments of word reading skills (pseudoword reading and sight word reading) were conducted with all participants in August (T1), October (T2) and January (T3) in Grade 2. See Table 3 for more detailed information. In addition, at T1, children were assessed with two additional tests; letter naming and PA. The outcome at T1 in pseudoword reading, sight word reading, letter naming and PA was used to individualize the content of the systematized phonics.

**TABLE 3:** Design of the study

Group 1	T1 (August)	Systematized phonics (30 school days, 30 min/day)	T2 (October)	Classroom instruction (30 school days, 30 min/day)	T3 (January)
Group 2		Classroom instruction (30 school days, 30 min/day)		*Systematized phonics* (30 school days, 30 min/day)	
Weeks	1–2	3–9^1^	10^2^	12–18^1^	22–23^3^

*Note*: 1 = Week 7 of intervention, time to catch up missed sessions, 2 = Week 11 autumn leave, 3 = week 19–21 Christmas holiday.

### 2.3 Design

A quasi‐experimental cross‐over design was used, with group as independent variable and post‐measures of pseudoword reading and sight word reading as dependent variables. Children in Group 1 were given systematized phonics individually one session/weekday (30 min/session) for 6 weeks during September and October in Grade 2 immediately after T1. During this period, Group 2 received regular classroom instruction. In November and December, Group 2 received the same amount and type of training as Group 1, immediately after T2. During this period, Group 1 received regular instruction in the classroom. The total intervention time for both groups was approximately 15 hr (2.5 hr per week). Due to the Christmas holiday, T3 was assessed 3 weeks after the last week of intervention/classroom instruction. See Table 3 for an overview of the design.

### 2.4 Measures in Grade 1

*Word reading*. Children were instructed to silently read chains of words where the blank space between words had been removed (Jacobson, 2014). Children marked each word boundary with a drawn line. Each word‐chain consisted of three semantically unrelated words (in total 80 word‐chains). The score was the number of correctly marked word boundaries within 2 min. Test–retest correlations were .89 for children in Grade 2 according to an earlier edition of the manual (Jacobson, 2001).

*Reading comprehension*. Children were instructed to silently read one or two sentences and to mark the correct picture corresponding to the content of the sentence (s) out of five alternatives (Järpsten, 2004). The score was the total number of correctly marked pictures within 7 min of reading. Maximum score was 20. Cronbach's alpha was .86 and test–retest reliability was .78 for Grade 1 according to the manual (Järpsten, 2004).

### 2.5 Individual assessments in Grade 2 at T1, T2 and T3

*Letter naming at T1*. Children were instructed to name 24 lower‐case letters and 24 upper‐case letters *(*Taube, Tornéus, & Lundberg, 1984). The letters were presented in rows in a random order. The total score was the sum of correct responses for both lower‐ and upper‐case letters (max 48).

*PA at T1*. PA was assessed with the subtests phoneme segmentation and phoneme blending (Taube et al., 1984). In phoneme segmentation, the child was asked to segment orally presented words into phonemes, for example, ‘lamp’ to l‐a‐m‐p (word length: four to seven phonemes). Test–retest reliability was .76 for Grade 1 and 2 according to the manual. In phoneme blending, the child was asked to blend orally presented phonemes to a word, for example, r‐e‐s‐t to ‘rest’ (word length: four to seven phonemes). Test–retest reliability was .70 for Grade 1 and 2 according to the manual. The total score for PA was the number of correct responses in both phoneme segmentation and phoneme blending (max 34).

*Pseudoword reading at T1*, *T2 and T3*. Children read pseudowords out loud (one to three syllables) from two different lists of words (A and B), as quickly and correctly as possible within 45 s for each word list (Elwér, Fridolfsson, Samuelsson, & Wiklund, 2013). The total score was the sum of correctly decoded words from the two lists of words (A and B). Maximum score was 126. Test–retest reliability for word‐list A and B were .81 and .73, respectively, in Grade 2 according to the manual.

*Sight word reading at T1*, *T2 and T3*. Children read words (one to four syllables) out loud as quickly and correctly as possible from two different lists of words (A and B) within 45 s for each word list (Elwér et al., 2013). The total score was the sum of correctly recognized words from the two lists of words (A and B). Maximum score was 200. Test–retest reliability for word‐list A and B were .93 in Grade 2 according to the manual.

### 2.6 Systematized phonics

Children received systematized phonics 30 min/day in a one‐to‐one setting together with a teacher for 6 weeks, totalling 15 hr. To increase fidelity, a seventh week was offered to reach the recommended instruction time in case of missed sessions. Each intervention session included exposure to the following three components in order:

1. Phoneme–grapheme correspondence
  - Naming letters in memory games or by reading lists of letters. The included letters were adjusted to each child's letter knowledge at T1.
2. Word recognition
  - Card games from Trugs (Häggström & Frylmark, 2010; Jeffrey, 2018) or reading lists of words from Bravkod (Jönsson, 2010). The length and phonological complexity of the included words were adjusted to each child's word reading level at T1.
3. Phonemic awareness
  - Games focusing on phoneme segmentation and blending.

In the section on word recognition (2), card games from Trugs were used 3 days/week and word lists from Bravkod were used 2 days/week. The level of instruction was adjusted according to the individual child's progress. When a child was able to read three‐letter words out loud, four‐letter words were introduced. Words with consonant‐vowel‐consonant (CVC) structure were introduced before words with CCV, making sure that each child managed to decode words with a simpler phonological structure before words with a more complex structure were introduced. The instruction followed a synthetic phonics approach. In the sections on phoneme–grapheme correspondence and PA, the content was individualized in relation to the outcome at the assessment at T1. This was to make sure each individual child received targeted phoneme–grapheme correspondence training and targeted complexity of the PA components. After 3 weeks of intervention, children's word reading level was evaluated to decide if reading on text‐level could be introduced as a fourth component in the intervention.

*Trugs*. Swedish Trugs consists of 10 reading stages and 2 boxes with word cards (Häggström & Frylmark, 2010). Short alphabetically spelled words constitute the first box and longer irregularly spelled words constitute the second. In the present study only box number 1, and Stages 1–5 were used (stage 1, CVC, 2, CVCV, 3, CCVC, 4, CVCC and 5, longer words with CCC in initial or medial position with maximum length 11 letters). Each stage includes four different games. The games have deliberately been made short to increase variety and maintain the players' interest and motivation. All words included in the games should be read aloud.

*Bravkod*. The objective of Bravkod is to automatize decoding skills through repeated reading (Eriksson, 2016). The material consists of reading lists with letters, syllables and high‐frequency words at the basic level, followed by words with complex phonological structure and irregular spelling at the advanced level. Following a playful warmup, the child reads lists of letters, syllables and words out loud, as quickly and accurately as possible. Thus, both phoneme–grapheme correspondence, sub‐word units and word units are trained.

### 2.7 Intervention procedure

Fourteen teachers participated in the intervention as instructors (specialist education teachers or teachers in primary school). The teachers participated in a lecture about word reading difficulties and early reading instruction as well as a workshop about the three different sections in the intervention (letter knowledge, word recognition and PA). Treatment fidelity was ensured through individual written lesson plans for each child composed by the project leader and the first author. The lesson plans specified which content the three sections should include. Content was based on the outcome from the individual assessment at T1 (August). Each lesson plan was discussed with the teachers before the start of the intervention. During the intervention, the teachers had two meetings/period with an experienced specialist education teacher (project leader) and one of the researchers (first author) for support, mainly regarding challenges in adjusting tasks to the correct level for each child. If a session could not take place, it was compensated for with extra sessions at the end of the intervention period.

### 2.8 Data analyses

All data are from assessments T1, T2 and T3. Analyses of skewness revealed that all dependent measures were less than ±0.69, with mean values within three standard errors, except for letter naming (−2.687) and PA (−1.442) at T1. Levene's test for the word reading scores were non‐significant, all *p*s > .17, indicating equal variance across groups for all the dependent measures at T1, T2 and T3. Effect sizes are reported for the within‐group variables from T1 to T2 and T2 to T3. Effect sizes are reported as Cohen's *d*; small effect = .2, medium effect = .5 and large effect = .8 (Cohen, 1988). A mixed design ANOVA was conducted in two sets to assess the impact of the intervention on the word reading scores related to time period of intervention (within‐subject effects) and group (between‐subject effects); in the first set of analyses from T1 to T2, and in the second set of analyses from T2 to T3. The two separate steps of mixed design ANOVA was chosen due to the cross‐over study design. The significance value was set at *p* < .05 for all comparisons. Effect sizes for the ANOVA are reported as partial eta squared (*ηp* ^*2*^; small effect = .01, medium effect = .06 and large effect = .138, Cohen, 1988).

## 3 RESULTS

### 3.1 Descriptive statistics for T1, T2 and T3 for Group 1 and Group 2

Table 4 presents all test scores at T1, T2 and T3. There were no significant differences between the groups at T1 for any of the measures using an independent samples *t*‐test (all *p*s > .21).

**TABLE 4:** Reading, letter naming and phonological processing scores at T1, T2 and T3

Measure	Group 1			Group 2		
	*n*	M	*SD*	*n*	M	*SD*
*T1 August*						
Letter naming	22	45.18	4.32	27	45.33	4.18
PA	22	26.27	6.67	27	24.04	8.21
Pseudoword reading (*z*‐scores)	22	20.18 (−1.26)	6.62 (0.36)	27	19.78 (−1.28)	7.93 (0.44)
Sight word reading (z‐scores)	22	29.41 (−1.56)	11.26 (0.34)	27	28.52 (−1.58)	13.25 (0.40)
*T2 October*						
Pseudoword reading (*z*‐scores)	22	34.55 (−0.47)	10.39 (0.57)	27	25.04 (−0.99)	8.49 (0.47)
Sight word reading (*z*‐scores)	22	46.50 (−1.04)	14.56 (0.44)	27	38.74 (−1.27)	14.54 (0.44)
*T3 January*						
Pseudoword reading (*z*‐scores)	22	34.36 (−0.48)	11.00 (0.60)	27	35.48 (−0.42)	9.15 (0.50)
Sight word reading (*z*‐scores)	22	53.95 (−0.81)	18.32 (0.55)	27	56.00 (−0.75)	17.59 (0.53)

*Note*: All reading scores are presented in raw scores with *z*‐values within parentheses.

Abbreviation: PA, phonemic awareness.

### 3.2 General effects of systematized phonics from T1 to T2 and from T2 to T3

General effects of the intervention are presented in Table 5. Both groups improved their pseudoword and sight word reading from T1 to T2 in raw scores (see Table 5). Large effect sizes were revealed for Group 1 in change between T1 and T2 in both pseudoword (*d* = 1.65) and sight word reading (*d* = 1.31) after systematized phonics while moderate effect sizes were revealed for Group 2 in pseudoword (*d* = 0.64) and sight word reading (*d* = 0.73) after classroom instruction.

**TABLE 5:** Effect sizes within group from T1 to T2 to T3 in reading measures

	T1		T2		Difference T1 and T2		T3		Difference T2 and T3	
	M	*SD*	M	*SD*	M (M_T2_ − M_T1)_	Cohen's *d* ^a^	M	*SD*	M (M_T2_ − M_T1)_	Cohen's *d* ^a^
*Pseudoword reading*										
Group 1 (*n* = 22)	20.18	6.62	34.55	10.39	14.37	1.65	34.36	11.00	−0.19	0.02
Group 2 (*n* = 27)	19.78	7.93	25.04	8.49	5.26	0.64	35.48	9.15	10.44	1.18
*Sight word reading*										
Group 1 (*n* = 22)	29.41	11.26	46.50	14.56	17.09	1.31	53.95	18.32	7.45	0.45
Group 2 (*n* = 27)	28.52	13.25	38.74	14.54	10.22	0.73	56.00	17.59	17.26	1.07

*Note*: Group 1 received systematized phonics and Group 2 received classroom instruction between T1 and T2. Group 2 received systematized phonics and Group 1 received classroom instruction from T2 to T3.

^a^ Cohen's *d* = (M at T2 − M at T1)/*S*_pooled,_ *S*_pooled_ = √(ST1^2^ + ST2^2^)/2.

Within‐group changes between T2 and T3 demonstrated an opposite pattern. Here, Group 2 improved their mean scores with large effect sizes in both pseudoword (*d* = 1.18) and sight word reading (*d* = 1.07), while Group 1 made no progress in pseudoword reading (*d* = 0.02) and a small improvement in sight word reading (*d* = 0.45) from T2 to T3 after classroom instruction.

In summary, both groups made larger improvements on both reading measures after systematized phonics (large effect sizes) than after classroom instruction (small to moderate effect sizes).

**FIGURE 1:**
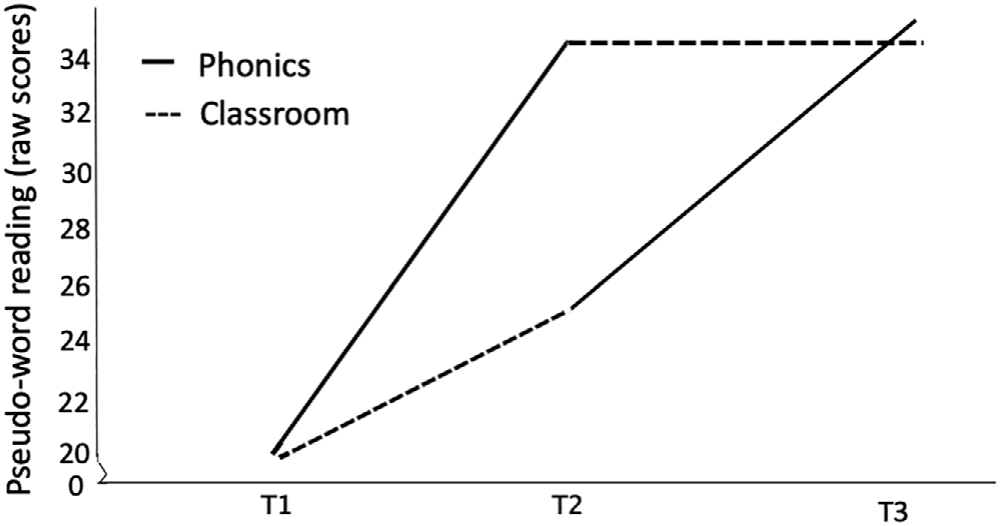
Pseudoword reading mean raw scores at test point 1, 2 and 3 after phonics training (solid line) and classroom instruction (dashed line). *Note*. Group 1 started with phonics training followed by classroom instruction, Group 2 started with classroom instruction followed by phonics training

**FIGURE 2:**
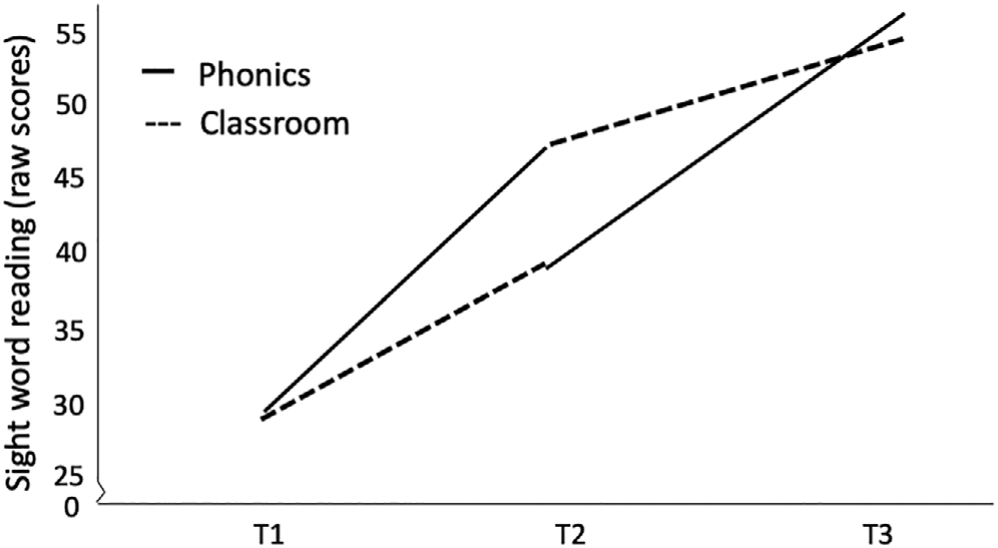
Sight word reading mean raw scores at test point 1, 2 and 3 after phonics training (solid line) and classroom instruction (dashed line). *Note*. Group 1 started with phonics training followed by classroom instruction, Group 2 started with classroom instruction followed by phonics training [Correction added on 07 October 2020, after online publication: The duplicate image of figure 2 has been removed in this version.]

See Figure 1 for pseudoword reading scores at T1, T2 and T3 and Figure 2 for sight word reading scores at T1, T2 and T3. For pseudoword reading, the mixed design ANOVA revealed a substantial main within‐subject effect of time *F*(1, 47) = 119.74, *p* < .001, *ηp* ^*2*^ = .72 and a medium main between‐subject effect of group, *F*(1, 47) = 4.84, *p* = .03, *ηp* ^*2*^ = .09 from T1 to T2. There was also a significant interaction between time and group, *F*(1, 47) = 25.78, *p* < .001, *ηp* ^*2*^ = .35. Thus, the pseudoword reading scores of Group 1 improved more with time than Group 2, with Group 1 having significantly higher scores at T2 than Group 2 (Group 1, *M difference* T1–T2 *=* 14.37, Group 2, *M difference* T1–T2 = 5.26).

For sight word reading, the mixed design ANOVA revealed a substantial main within‐subject effect of time, *F*(1, 47) = 149.95, *p* < .001, *ηp* ^*2*^ = .76, but there was no main between‐subject effect of group, *F*(1, 47) = 1.35, *p* = .25, *ηp* ^*2*^ = .03. There was a significant interaction effect between time and group, *F*(1, 47) = 9.48, *p* = .003, *ηp* ^*2*^ = .17. This indicates that both groups improved their scores in sight word reading from T1 to T2 but that the changes in sight word reading were mainly attributed to Group 1 (Group 1, *M difference* T1–T2 *=* 17.09, Group 2, *M difference* T1–T2 = 10.22).

Following this, the corresponding mixed design ANOVA from T2 to T3 showed that for pseudoword reading, there was a main within‐subject effect of time, *F*(1, 47) = 31.45, *p* < .001, *ηp* ^*2*^ = .40, but no main between‐subject effect of group *F*(1, 47) = 2.54, *p* = .12, *ηp* ^*2*^ = .05. A significant interaction effect was revealed between time and group, *F*(1, 47) = 33.72, *p* < .001, *ηp* ^*2*^ = .42. The main effect of time was driven by the progress in Group 2 suggesting an effect of the systematized phonics (group 1, *M difference* T2–T3 *=* ‐0.19, Group 2, *M difference T2–T3* = 10.44).

For sight word reading, there was a substantial main within‐subject effect of time *F*(1, 47) = 142.70, *p* = <.001 *ηp* ^*2*^ = .75, but no main between‐subject effect of group, *F*(1, 47) = .39, *p* = .54, *ηp* ^*2*^ = .008. There was, however, a significant interaction effect between time and group, *F*(1, 47) = 22.46, *p* < .001, *ηp* ^*2*^ = .32. This indicates that both groups improved their scores in sight word reading from T2 to T3, but that the changes in sight word reading were mainly attributed to Group 2 suggesting again an effect of the systematized phonics (Group 1, *M difference* T2–T3 *=* 7.45, Group 2, *M difference* T2–T3 = 17.26).

### 3.3 Percentage of children with age‐adequate reading skills at T1 and T3

**FIGURE 3:**
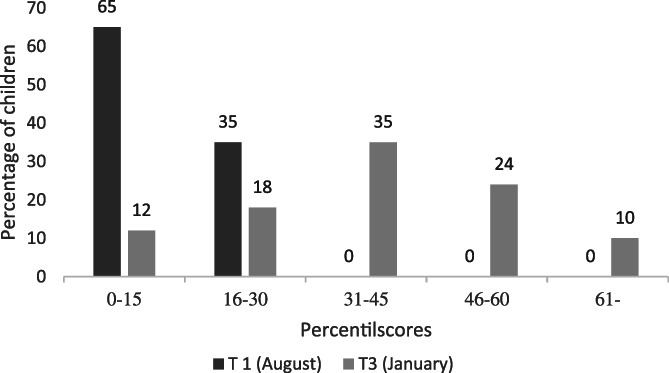
Percentage of children (*n* = 49) performing under and above percentile 15 in pseudoword reading at pre‐intervention (T1) and post‐intervention (T3)

At pre‐intervention (T1), 65% of the children (Group 1 and 2 collapsed) performed at or below percentile 15 in pseudoword reading. At post‐intervention (T3), when both groups had completed the systematized phonics, only 12% of the children still performed at or below percentile 15. As can be seen in Figure 3, none of the children performed above percentile 30 at pre‐intervention (T1) compared to 69% at post‐intervention (T3).

**FIGURE 4:**
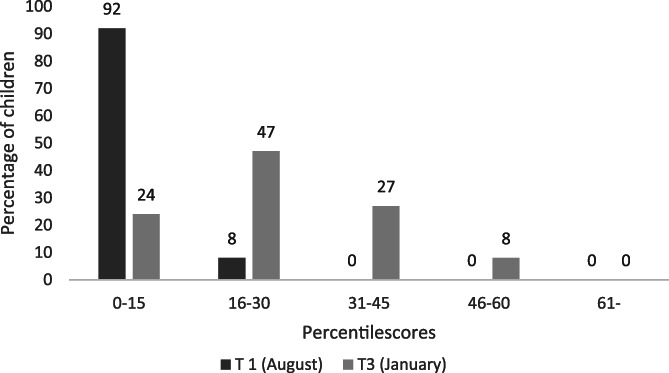
Percentage of children (*n* = 49) performing below and above percentile 15 in sight word reading at pre‐intervention (T1) and post‐intervention (T3)

At pre‐intervention (T1), 92% of the children performed at or below percentile 15 on sight word reading. At post‐intervention (T3), when both groups had completed the systematized phonics only 24% of the children still performed at or below percentile 15. As can be seen in Figure 4, none of the children performed above percentile 30 at pre‐intervention (T1) compared to 35% at post‐intervention (T3).

## 4 DISCUSSION

The present study aimed to explore whether systematized phonics improved word reading skills in 49 Swedish second grade children identified as poor readers at the beginning of Grade 2, and to what extent age‐typical reading was reached at post intervention. The study had a cross‐over design exploring within‐ and between‐group effects during the two different conditions: systematized phonics and classroom instruction. Both groups received approximately 15 hr of systematized phonics in a one‐to‐one setting over 6 weeks.

### 4.1 General effects of systematized phonics from pre‐ to post‐test in word reading (T1–T2–T3)

Analysis of change in raw scores showed that both groups increased their word reading skills from T1 to T2, but Group 1 outperformed Group 2 with large effect sizes in both pseudoword reading and sight word reading, thus confirming a positive effect of systematized phonics. At T3, when Group 2 had received systematized phonics, they outperformed Group 1 in a comparable manner. This is in line with previous studies reporting positive reading outcomes for intervention methods with a primary focus on PA and phoneme–grapheme correspondence (Gustafson et al., 2011; McArthur et al., 2018; Vellutino et al., 2008; Wanzek et al., 2018; Wolff, 2011; Wolff, 2016). Gustafson et al. (2011) compared computer‐based reading intervention (bottom‐up, top‐down or a combination of both) in Swedish second grade children, and showed that the combined training resulted in large effect sizes for pseudoword reading, sight word reading and word decoding. The present study observed comparably higher effect sizes for pseudoword reading. Notably, greater focus was placed on children reading syllables and words out loud compared to the study by Gustafson et al., which focused more on comprehension skills and phoneme identification.

More recently, McArthur et al. (2018) reviewed systematized phonics from 14 different studies in English speaking children and adolescents. The mean intervention score for irregular words, a measure of accuracy in sight word reading, indicated a large effect (four studies). The mean intervention non‐word fluency score, a measure of pseudoword reading, indicated a small effect (three studies). Compared to McArthur et al., larger effect sizes were observed in the present study, suggesting that particular elements of Bravkod and Trugs may have been beneficial for word recognition skills in a semi‐transparent orthography. This difference could be due to the different patterns of early reading development in deep and transparent orthographies. In deep orthographies, children need more time to achieve fluent word recognition skills compared to transparent orthographies (Seymour, Aro, & Erskine, 2003). Consequently, systematized phonics may have a larger impact on children's word recognition skills in a semi‐transparent orthography such as Swedish, compared to in a deep orthography like English.

Lastly, in Wolff's study (Wolff, 2011), 9‐year‐old children took part in a multi‐component reading intervention (PA, reading fluency and comprehension; 12 weeks for a total of 40 hr). Although the children trained for a considerably longer time than in the present study, only small effect sizes were observed, in this case, for PA and reading speed. This could be due to the multi‐component approach in Wolff's intervention study, leaving less time for systematized phonics. However, it is noteworthy that Wolff observed that the effects were still present at a 1 year follow‐up, an element that was not part of the design of the present study.

Both Bravkod (Jönsson, 2010) and Trugs (Häggström & Frylmark, 2010; Jeffrey, 2018) are materials used in special education in Swedish school settings, but to the authors' knowledge, this is the first time they have been evaluated in a quasi‐experimental design. Both materials follow a synthetic phonics approach, including words of varying length and phonological complexity, making it possible to individualize types of words used according to each child's progress in the intervention.

Although significant effects were found for both pseudoword and sight word reading, an advantageous effect was seen for pseudoword reading. This may be a result of the instructions' focus on phoneme–grapheme correspondence, a crucial skill in pseudoword reading, while sight word reading may be influenced by other skills as well, for example, vocabulary, rapid automatic naming and morphological knowledge (McArthur et al., 2015; Murray, McIlwain, Wang, Murray, & Finley, 2019). Importantly, Bravkod includes elements at the sub‐lexical level which may have supported children's orthographic reading of transparent words. Recognition of high‐frequency ‘base words’ possibly support decoding of longer seemingly unfamiliar words (Carlisle & Stone, 2005; Elbro & Arnbak, 1996).

### 4.2 Percentage of children with age‐adequate reading skills at post‐intervention (T3)

The finding that almost twice as many children improved their pseudoword reading scores as compared to their sight word reading scores reconfirms that systematized phonics had greater effect on pseudoword reading than sight word reading. Improved skills in pseudoword reading may contribute to further progress in sight word reading as well as in text reading, since children can use their newly acquired skills to uncover new orthographic patterns (Share, 1995). However, such transfer effects could not be confirmed in the present study. After systematized phonics, Group 1 made significant progress in pseudoword reading but no transfer effects in relation to sight word reading were identified during the following period of classroom instruction. This is in line with McArthur et al. (2015) and the meta‐review by Suggate (2016), where limited generalization to broader reading skills was observed after systematized phonics. This implies that more longitudinal studies are needed to investigate more explicitly to what extent systematized phonics has an effect on broader reading skills, and whether other elements, such as well‐structured reading instruction in the classroom, are required for long‐term improvements in word reading (see review by Slavin, Lake, Davis, & Madden, 2011). It is noteworthy though, that three out of four elements that Slavin et al. identified as effective in reading programs were incorporated in the present study: one‐to‐one tutoring, qualified teachers and phonics.

In addition to identifying the crucial elements for effective early reading instruction, it is also important in a school setting to find models where children with weak reading skills can be identified early, in order to get adequate support. In the United States, a response to intervention model (RTI) has been implemented in many schools with good outcome (Fuchs & Fuchs, 2006). In an RTI‐framework, well‐structured interventions are given to all children identified with reading difficulties, at first in small groups and thereafter individually to children with persisting difficulties (Catts, Nielsen, Bridges, Liu, & Bontempo, 2015; Capellini, César, & Germano, 2015; Partanen & Siegel, 2014; Vellutino et al., 2008.). Poor RTI outcomes has been identified as a key‐aspect in finding children in need of more longstanding support and in identifying children who may fulfil the criteria for dyslexia (Vellutino et al., 2008).

In Sweden, RTI has not been used to a large extent despite research findings supporting the implementation of individualized phonics intervention before considering a dyslexia diagnosis (Elliott & Grigorenko, 2014; Tunmer & Greaney, 2010). The results in this study support an RTI model, considering the substantial progress many children made from word reading skills below percentile 15 (an often‐used cut‐off for a dyslexia diagnosis) to scores above percentile 30 after the intervention. Considering the often very restrained resources for assessment and special needs support, using an RTI‐framework may also become an important tool in a Swedish school setting in order to identify children that require further assessment and support by specialists within the School Health Services.

### 4.3 Considerations

This study was carried out in a regular school setting. Unfortunately, it was not possible to randomize the assignment of children to different groups, due to organizational circumstances at the participating schools. On the other hand, there were no significant differences in reading scores between the two groups at pre‐test (T1), indicating similar reading levels at the start of the study. The cross‐over design also enabled the exploration of within‐group effects during the two different conditions: systematized phonics and classroom instruction.

A control group receiving a different type of word reading instruction would have strengthened the interpretation of the outcome. In the present study design, there is also a chance that the positive intervention effects to some extent are due to the novelty of the material as well as to receiving one‐to‐one attention compared to a classroom context where this is typically not the case. Including classroom‐based instruction in phonics as one of the conditions in the study would have been another option to control for the effects of one‐to‐one attention in the outcome.

Field observations of the implementation of the intervention would have strengthened the fidelity of the intervention procedure and content, but this was not possible due to limited resources. On the other hand, it is promising that the instructional elements provided to the teachers still resulted in a positive outcome for the intervention. In a regular school setting, it is not possible to maintain frequent supervision and observations when special needs support is implemented. Therefore, the present study serves as an example of an intervention program that is standardized enough to be implemented by teachers without a special needs background.

### 4.4 Implications for education and future research

The positive outcome in word reading skills after implementing systematized phonics for struggling second grade readers suggests that this model should be included as standard in early reading instruction in Sweden. Considering the substantial decrease in students performing below percentile 15 after 6 weeks of systematized phonics, this study also suggests the importance of providing well‐structured systematized phonics before considering a dyslexia diagnosis.

In future studies, there is a need to explore the long‐term effects of systematized phonics when it comes to broader reading skills such as sight word reading and reading fluency at text‐level. It would also be of great value to implement phonics in a classroom setting or small groups, since that is uncommon in a Swedish school setting and has to the authors' knowledge not been evaluated in any previous studies. Including measures of reading fluency at text‐level and delayed post‐tests (1 year or more) would have strengthened the contribution to the research field in this study.
